# Effects of Adolescent Empathy on Emotional Resilience: The Mediating Role of Depression and Self-Efficacy and the Moderating Effect of Social Activities

**DOI:** 10.3390/bs14030228

**Published:** 2024-03-12

**Authors:** Jun Wang, Quanqi Yang, Xian Yu, Li Hu

**Affiliations:** 1 CAS Key Laboratory of Mental Health, Institute of Psychology, Chinese Academy of Sciences, Beijing 100101, China; wangjun1@cbnet.com.cn (J.W.); yangqq@psych.ac.cn (Q.Y.); 2 Department of Psychology, University of Chinese Academy of Sciences, Beijing 100101, China; 3 Faculty of Humanities, Changzhou Vocational Institute Textile and Garment, Changzhou 213164, China

**Keywords:** empathy, emotional resilience, depression, self-efficacy, social activities

## Abstract

Background: This study aims to investigate the role of empathy in fostering emotional resilience and the impact of social activity on adolescents’ mental health. Methods: A survey was conducted on 1666 adolescents aged from 11 to 15 across seven cities in China, including Beijing, Shanghai, etc. Results: Empathy is significantly positively related to emotional resilience (*β* = 0.128; *p* < 0.001). Moreover, depression acts as a mediator between empathy and emotional resilience (*ab* = 0.106, *SE* = 0.021, 95% confidence interval [0.065, 0.146]), while self-efficacy plays an incomplete mediating role (*ab* = 0.286, *SE* = 0.020, and a 95% confidence interval of [0.246, 0.326]). Additionally, social activity was able to modulate the relationship between empathy and depression (*β* = 0.004; *p* < 0.001), as well as the relationship between empathy and self-efficacy (*β* = 0.003; *p* < 0.001). Conclusion: For adolescents, (1) greater emotional resilience is associated with higher levels of empathy; (2) improving empathy can indirectly enhance their emotional resilience by reducing their depression level; (3) the relationship between empathy ability and depression is modulated by social activity, and the predictive effect of empathy on depression is reduced when adolescents have high social activity levels; and (4) the relationship between empathy and self-efficacy is also modulated by social activity, and the predictive effect of empathy on self-efficacy is enhanced when adolescents have higher levels of social activity.

## 1. Introduction

The teenage years are an important time for emotional development [1]. Researchers have found that emotional resilience can serve as a protective resource for children and adolescents, reducing their psychological risk during stress or major life changes [2].

Emotional resilience, derived from psychological resilience, denotes an individual’s capacity to cultivate positive emotions and rebound from negative emotions in response to stress [3]. Research has demonstrated a close association between emotional resilience and negative mental health indicators, highlighting it as a critical factor in mental well-being. According to Zhu [4], low emotional resilience underlies many mood disorders. Davidson [5] suggested that individuals with low emotional resilience are more susceptible to negative emotions when facing challenges. Conversely, those with higher emotional resilience demonstrate greater resistance and better recovery from stress [6]. The study conducted by Dolgova and Rokitskaya [7] emphasized the adaptive potential inherent in emotional resilience, enabling individuals to balance internal emotional states and external environmental factors, thus enhancing problem-solving abilities during stressful situations. Hence, it can be inferred that improving emotional resilience helps individuals perform better under stress. Emotional resilience is a complex and multifaceted structure [8], with factors including accurate empathy, self-efficacy, good social support, etc. [9]. As one of the related factors of emotional resilience, the role of empathy in improving emotional resilience and mental health deserves in-depth discussion.

### 1.1. Relationship between Empathy and Emotional Resilience

Empathy refers to an individual’s ability to understand the situation of others, perceive or imagine their emotional state, experience their feelings, and respond appropriately [10,11,12]. Since the concept of empathy was proposed, the definition of its components has evolved from a one-dimensional to a multi-dimensional construct. Although there is currently no clear consensus in the academic community regarding the components of empathy, most scholars suggest that empathy includes at least emotional empathy and cognitive empathy [13,14]. Emotional empathy is manifested as the ability to spontaneously experience alternative emotional experiences for the emotions felt by others, while cognitive empathy is demonstrated as the ability to recognize the emotions of others and understand their perspectives [15]. The teenage years are a particularly critical time for the development of empathy [16]. Researchers studying the lifelong development of empathy have found that emotional empathy tends to stabilize from adolescence to adulthood [17], and cognitive empathy also develops to a new level in adolescence, with the ability to empathize with abstract groups and produce prosocial behaviors [18]. Empathy is an important life skill related to mental health and positive adjustment [19,20]. Good empathy helps individuals establish harmonious interpersonal relationships, thereby positively impacting mental health [21].

The literature has noted that emotional resilience and empathy are interconnected in terms of their components. Abolin [22] stated that emotional resilience comprises the interaction of emotional, cognitive, and behavioral components. Other researchers have found that emotional resilience is a comprehensive attribute of a person, which is characterized by the interaction of emotion, cognition, behavior, and volitional components [23]. When considering the emotional and cognitive components of empathy, it becomes evident that emotional resilience and empathy are, at the very least, connected in terms of cognition and emotion. Grant and Kinman [9] further proposed that accurate empathy can enhance emotional resilience. Consequently, this study posits the hypothesis that empathy can be related to emotional resilience at both the cognitive and emotional levels.

### 1.2. The Mediating Role of Depression

From the emotional perspective, this study selected depression as the mediating factor. Depression is characterized by feelings of sadness, despondency, or low spirits and represents a common type of negative emotional state [24]. Depression, as a negative emotion, has become a major risk factor affecting the physical and mental health of adolescents in the 21st century [25]. It is closely related to empathy [18] and also to emotional resilience [26].

There is a connection between empathy and depression in their conceptual structures [27]. Empathy reflects an individual’s ability to recognize and understand emotions [28], and depression is closely related to emotional processing ability [29]. From the perspective of the relationship between empathy and depression, Shi et al. [30] stated that fostering empathic skills can improve psychological health and, consequently, decrease the likelihood of depression. In a study of adolescents, Chen [31] found that perspective-taking in empathy was significantly negatively correlated with depression, and empathic concern was significantly negatively correlated with depression. In a large-sample study, Benik et al. [32] found a linear relationship between cognitive empathy levels and depressive symptoms, and individuals with lower levels of cognitive empathy exhibited more depressive symptoms. Through net analysis, Li et al. [33] found that different dimensional constructs of empathy were negatively associated with different symptoms of depression. For example, the people with low empathic concern have more suicidal thoughts and psychomotor agitation or retardation, and the people with low perspective-taking have higher level of concentrating. Similar to these results, a meta-analysis [34] that included 37 studies showed that depression is also associated with limited cognitive empathy, such as poor perspective-taking, theory of mind, and empathic accuracy.

From the perspective of depression and emotional resilience, various studies [35,36] have identified a negative correlation between emotional resilience and anxiety and depression, indicating that individuals with higher levels of anxiety and depression typically exhibit lower emotional resilience. Wu et al. [37] found that depressed individuals with anhedonia experienced lower mean positive emotions. As an important component of emotional resilience [38], positive emotional ability can make individuals better at applying positive emotions to negative situations, thereby adapting to these situations positively [39]. This directly affects emotional resilience. In a study on middle school students, Pei et al. [40] found that there was also a significant negative correlation between self-rated depression scores and self-rated emotional flexibility scores. Thus, we assumed that empathy is negatively related to depression and that depression is negatively related to emotional resilience, so depression could mediate the relationship between empathy and emotional resilience.

### 1.3. The Mediating Role of Self-Efficacy

Self-efficacy, a crucial component of Social Cognitive Theory, refers to an individual’s belief in their competence to execute tasks and the confidence in their ability to perform specific actions [41]. As an important cognitive factor, self-efficacy is significant in improving mental health [42,43]. It is closely related to empathy [44] and a key factor affecting emotional resilience [26].

Regarding the cognitive dimension, researchers have found that adolescent cognitive empathy is positively related to self-efficacy [45]. Ruan et al. [46] validated a significant positive correlation between empathy and social self-efficacy. A study focusing on university students indicated that higher levels of empathy among students are associated with stronger self-efficacy [47].

Similarly, researchers have found self-efficacy to be a contributing factor to emotional resilience [9,48], which was verified in a study by Zhang et al. [49]. Individuals with high levels of self-efficacy experience more positive emotions in their work, while those with low self-efficacy often face more negative emotional experiences [50]. Thus, we assumed that empathy is positively related to self-efficacy, self-efficacy is positively related to emotional resilience, and self-efficacy could mediate the relationship between empathy and emotional resilience.

### 1.4. The Moderating Role of Social Activities

Previous research [51,52,53] demonstrates a significant correlation between empathy and social activities. Fu et al. [54] pointed out that individuals with higher empathy are more adept at perceiving emotions and, thus, are more likely to receive help and support from friends, family, and society. In a study performed on nursing staff, Cai et al. [55] suggest that the more support that nurses receive from their families, the higher their empathy levels. In addition, social group activities, as one of the ways to obtain social support, are closely related to empathy. This was also suggested by Wu et al. [56], who show a strong link between long-term ballroom dance training and empathy.

According to the social support theory, individuals obtain visible and invisible support through social connections, which can relieve psychological pressure, eliminate individual psychological barriers, and improve mental health [57]. Zhen et al. [58] found that a higher level of perceived social support has a greater impact on an individual’s psychological health and can also reduce stress and depression. Therefore, it is easy to infer that social support positively influences individuals’ emotions. Similarly, social group activities are closely related to obtaining social support and positive psychology. Researchers have found that a lack of social group activities can lead to an increase in students’ depression rates [59]. A study on children and adolescents in China during the COVID-19 pandemic found that children and adolescents who were required to attend online classes at home, minimize or stop outdoor activities, and avoid group social interactions were more likely to experience frequent feelings of anxiety and depression [60]. In contrast, social group activities can promote the formation of positive psychology in students [61]. Thus, we assumed that social group activities could play a moderating role between empathy and depression.

As for the relationship with self-efficacy, previous research has shown that participating in club activities has a positive impact on the development of self-efficacy. Female participants generally show higher enthusiasm for club activities than male participants. Furthermore, women’s self-efficacy tends to increase faster in the early stages of participating in these activities [62]. Wang et al. [63] found a direct link between the frequency of college student participation in club activities and their level of self-efficacy. More frequent participation is associated with higher self-efficacy. Therefore, we hypothesize that social group activities also play a moderating role in empathy and self-efficacy.

## 2. Materials and Methods

### 2.1. Methods

This study’s participants were selected from seven cities in China, including Beijing, Shanghai, Chengdu, etc. We initially surveyed 1807 middle and elementary school students using cluster random sampling. After excluding questionnaires that were highly repetitive or completed in less than half the allotted time, we obtained 1666 valid responses. The age range of the included sample was 11–15, with a mean age of 12.777 and a standard deviation of 1.138. The gender distribution included 812 males and 854 females. Of these participants, 526 were only children, while 1140 had siblings.

### 2.2. Materials

#### 2.2.1. Adolescent Emotional Resilience Questionnaire (AERQ)

The questionnaire created by Zhang et al. [64] is designed to evaluate the emotional resilience of adolescent students between the ages of 11 and 20 years. It consists of eleven items and functions as a self-report tool, requiring participants to respond based on personal experiences. The questionnaire utilizes a six-point Likert scale for responses, with options ranging from 1 (completely disagree) to 6 (completely agree). Notably, items 2, 5, 7, 10, and 11 are reverse-scored. The aggregate score derived from the responses indicates the respondents’ emotional resilience. A higher cumulative score signifies better emotional resilience. This instrument has been validated for its reliability and effectiveness in assessing adolescents’ emotional resilience. Our study recorded Cronbach’s α coefficient for the AERQ at 0.82.

#### 2.2.2. QCAE Empathy Scale for Chinese Adolescents (QCAECSA)

The questionnaire revised by Wang et al. [65] for Chinese adolescents aged 11–15 comprises seventeen items. It employs a four-point Likert scale for scoring, with options ranging from 1 (strongly disagree) to 4 (strongly agree). The score for each dimension is calculated by summing the scores of its corresponding items, and the total of all dimension scores determines the overall score of the scale. In our study, Cronbach’s α coefficient for the QCAECSA was 0.88.

#### 2.2.3. General Self-Efficacy Scale (GSES)

The questionnaire compiled by Schwarzer et al. [66] for ages older than 12 is unidimensional and includes ten items. It utilizes a four-point Likert scale for scoring, with options ranging from 1 (quite agree) to 4 (strongly agree). A higher total score on this scale reflects a stronger general sense of self-efficacy. Chu et al. [67] and An et al. [68] used this scale in adolescent research and confirmed its validity. Our study determined that Cronbach’s α coefficient for the GSES was 0.92.

#### 2.2.4. A Short Chinese Version of the Center for Epidemiologic Studies Depression Scale (CES-D)

Radloff’s Center for Epidemiological Studies Depression Scale (CES-D) was revised by He et al. [69] for the general population of China, aged 11–100; it comprises nine items and employs a four-point Likert scale for scoring. The scale ranges from 0 (‘none’) to 3 (‘most of the time’), with higher scores indicating a more pronounced level of individual depression. Cronbach’s α coefficient of the CES-D is 0.90.

#### 2.2.5. Social Activity Participation Scale (SAPS)

This scale, developed by Tang et al. [70], consists of nineteen items and employs a five-point Likert scale for scoring, with options ranging from 1 (strongly disagree) to 5 (strongly agree). Higher scores on this scale signify more active participation in social activities. The SAPS was originally designed for college students and used for adolescents. Yuan et al. [71] used this scale to measure the social participation level of Generation Z teenagers, confirming its validity. This study also used this scale to measure adolescents’ levels of social activity. In our study, Cronbach’s α coefficient for this scale remained at 0.89.

## 3. Results

### 3.1. Common Method Bias Test

To assess common method bias, we utilized the Harman single-factor test [72]. It involved conducting an exploratory factor analysis without rotation on all items across the four scales used in the study. The analysis yielded eight common factors, each with eigenvalues exceeding 1. The first common factor accounted for only 26.414 of the total variance. This result falls below the 40% threshold [73], indicating that common method bias is not a significant concern in our study.

### 3.2. Analysis of Empathy, Emotional Resilience, Depression, Self-Efficacy, and Social Activity Participation

The relationships between the variables in our study were quantified using Pearson’s correlation coefficients, as detailed in Table 1. We found that empathy in adolescents is significantly negatively correlated with depression (*r* = −0.142, *p* < 0.001) and significantly positively correlated with self-efficacy, emotional resilience, and social activities. Depression was significantly and negatively related to self-efficacy, emotional resilience, and social activity. Self-efficacy is significantly positively correlated with emotional resilience and social activities. Emotional resilience was significantly positively related to social activities.

### 3.3. The Mediating Role of Depression

**Figure 1:**
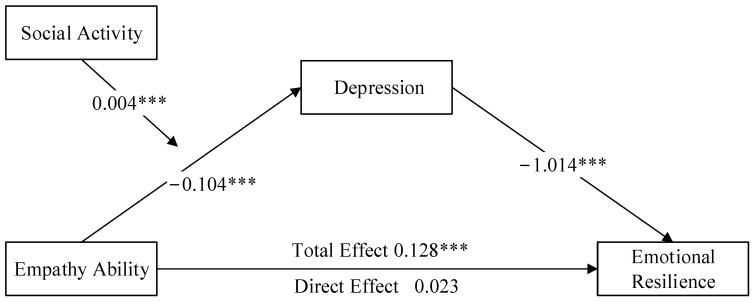
The mediating role of depression between empathy and emotional resilience and the regulation of social activity. Note: *** *p* < 0.001.

In this study, we initially utilized Model 4 of the PROCESS plugin in SPSS 26 to examine the mediating effect of depression on the relationship between empathy and emotional resilience. Refer to the study by Zhao et al. [74]. After controlling variables such as age, gender, and only-child status, according to the results in Table 2, model M1 shows that empathy is positively related to emotional resilience (*β* = 0.128; *p* < 0.001). Model M2 reveals that empathy significantly and negatively predicts depression (*β* = −0.104; *p* < 0.001). In Model M3, with both empathy and depression included, empathy no longer significantly predicts emotional resilience (*β* = 0.023; *p* > 0.05). In contrast, depression shows a significant negative prediction of emotional resilience (*β* = −1.014; *p* < 0.001). Bootstrap analysis confirms the significant mediating role of depression between empathy and emotional resilience, with a mediating effect of *ab* = 0.106, *SE* = 0.021, and a 95% confidence interval of [0.065, 0.146] (detailed in Table 3). These findings indicate that improving empathy in adolescents can enhance emotional resilience by decreasing depression levels (as shown in Figure 1).

### 3.4. The Moderating Role of Social Activity on Empathy and Depression

Employing Model 7 of the PROCESS plugin, we tested a moderated mediation model while accounting for variables such as age, gender, and only-child status. Model M7 (shown in Table 4) indicates that, when including empathy, social activity, and their interaction term, the R^2^ value results in 0.233, demonstrating a good model fit. The interaction between empathy and social activities significantly predicts depression (*β* = 0.004; *p* < 0.001). This finding suggests that social activity plays a moderating role in the relationship between empathy and depression (as shown in Figure 1).

**Figure 2:**
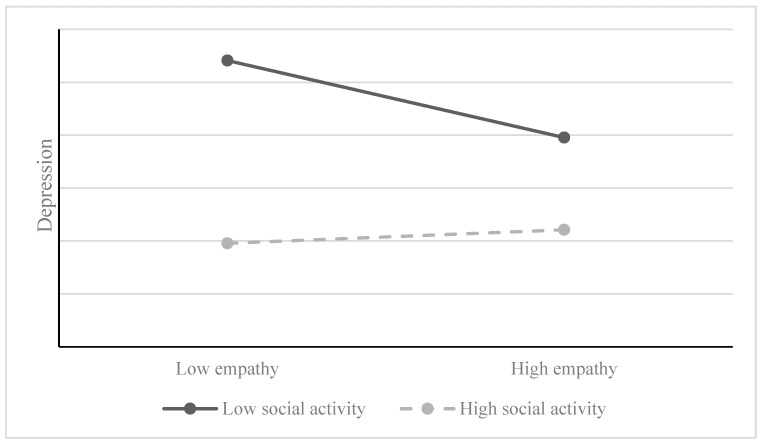
Relationship between empathy and depression in different levels of social activity.

To better illustrate the moderating role of social activity, our study classified participants into high and low social activity groups based on the mean level of social activity, adjusted by, plus or minus, one standard deviation. We then constructed a moderation diagram to visualize this relationship, as shown in Figure 1. Simple slope analysis revealed that, at low levels of social activity, empathy significantly and negatively predicts depression (*β* = −0.082, *t* = −2.081, and *p* < 0.05). Conversely, at high levels of social activities, empathy does not significantly predict the occurrence of depression (*β* = 0.038, *t* = 0.725, and *p* > 0.05) (as shown in Figure 2).

### 3.5. The Mediating Role of Self-Efficacy

**Figure 3:**
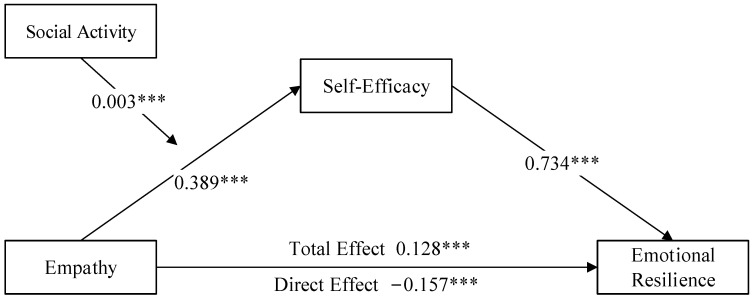
The concealment effect of self-efficacy between empathy and emotional resilience and the moderation of social activities. Note: *** *p* < 0.001.

We investigated the mediating role of self-efficacy in the relationship between empathy and emotional resilience. According to the findings presented in Table 5, after adjusting for age, gender, and only-child status, Model M4 showed that empathy significantly positively impacts emotional resilience (*β* = 0.128; *p* < 0.001). Model M5 indicated that empathy significantly and positively predicts self-efficacy (*β* = 0.389; *p* < 0.001). In Model M6, with both empathy and self-efficacy included, empathy was found to predict emotional resilience negatively and significantly (*β* = −0.157; *p* < 0.001), while self-efficacy shows a significant positive prediction of emotional resilience (*β* = 0.734; *p* < 0.001). The bootstrap analysis confirmed the significant mediating effect of self-efficacy between empathy and emotional resilience, with a mediating effect value of *ab* = 0.286, *SE* = 0.020, and a 95% confidence interval of [0.246, 0.326] (detailed in Table 6). These results suggest that improving empathy in adolescents can enhance emotional resilience by fostering self-efficacy. The direct effect (*c*’ = −0.157) and the mediating effect (*ab* =0.286) are in opposite directions, indicating that the path effect from empathy to emotional resilience with self-efficacy mediation counteracts the original negative direct effect in this mediation model, thus leading to a positive overall effect (as shown in Figure 3).

### 3.6. The Moderating Effect of Social Activity on Empathy and Self-Efficacy

Using Model 7 of the PROCESS plugin, after adjusting for variables such as age, gender, and only-child status, we examined the moderating effect of social activity on the relationship between empathy and self-efficacy. Model 8, presented in Table 7, reveals that including empathy, social activity, and their interaction term resulted in an R^2^ value of 0.380, indicating a good model fit. The interaction between empathy and social activity significantly predicted self-efficacy (*β* = 0.003; *p* < 0.001). This suggests that social activity is crucial to moderating the relationship between empathy and self-efficacy (as shown in Figure 3).

**Figure 4:**
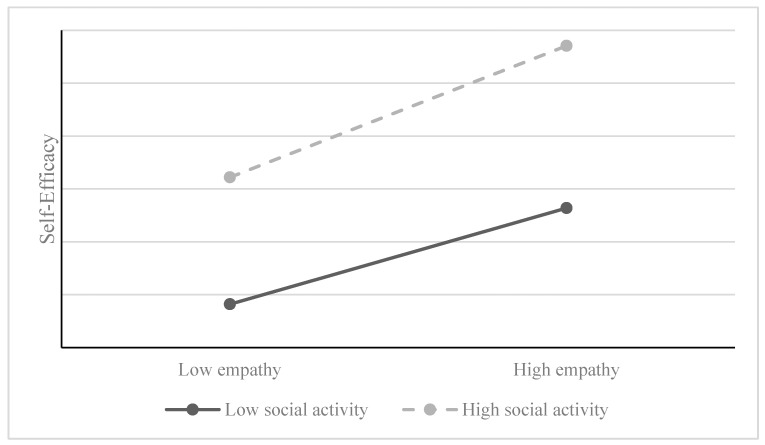
Relationship between empathy and self-efficacy in different levels of social activity participants.

Simple slope analyses (Figure 4) showed that, at low levels of social activities, empathy significantly and positively predicts self-efficacy (*β* = 0.273; *t* = 7.323, and *p* < 0.001). At high levels of social activities, empathy not only continues to significantly and positively predict self-efficacy but the predictive effect is amplified *(β* = 0.323; *t* = 6.339, and *p* < 0.001).

## 4. Discussion

This study investigated the relationship between empathy and emotional resilience in adolescents, revealing a connection between the two at the emotional level (depression) and the cognitive level (self-efficacy). Additionally, by assessing the moderating effect of social activity, this study clarified the boundary conditions that govern the interaction between social activities, empathy, depression, and self-efficacy. Our results provide valuable guidance for educators aiming to enhance adolescents’ emotional resilience and self-efficacy while mitigating depressive emotions.

### 4.1. The Relationship between Empathy and Emotional Resilience

Through this study, we found that empathy can be directly related to emotional resilience and indirectly related to emotional resilience through depression and self-efficacy. The results of this study show that there is a positive correlation between empathy and emotional resilience, indicating that developing individual empathy can improve individual emotional resilience within a certain range, which is consistent with the results of Liu et al. [75] and Arsini et al. [76]. Our results also partially prove Grant et al.’s factor model of empathy [77]. Good empathy is an important factor in emotional resilience, and empathy can enhance emotional resilience.

### 4.2. The Mediating Role of Depression

Our study identified how depression serves as a full mediator between empathy and emotional resilience. Increased empathy leads to reduced depression and a corresponding rise in positive emotions, thereby bolstering emotional resilience. Conversely, we observed a negative correlation between empathy and depression in adolescents in our results, which aligns with previous research [24,30,78]. Cherewick et al. [79] found in their study that adolescents with higher empathy reported lower psychological internalizing and externalizing symptoms, such as anxiety, depression, high aggression, etc. This shows that empathy plays a certain protective role and is of great significance to the physical and mental development of adolescents. However, empathy does not always play a positive role. Excessive empathy may increase the risk of depression [80,81]. This may be related to whether teenagers themselves have complete emotional regulation abilities [80].

### 4.3. The Mediating Role of Self-Efficacy

Through mediating effect analysis, this study found that empathy can have a great positive impact on emotional resilience through self-efficacy. Adolescents’ empathy plays a crucial role in improving self-efficacy [82]. Similarly, self-efficacy can also help adolescents overcome adversity [83], thereby improving emotional resilience. This is consistent with Zhang’s study [84]. Additionally, self-efficacy plays a masking effect in the relationship between empathy and emotional resilience. Emotional resilience is significantly positively correlated with self-efficacy. However, the total effect size is smaller than the mediation effect size of self-efficacy, suggesting the presence of other negative mediating pathways between empathy and emotional resilience. These pathways, along with self-efficacy, play a crucial bridging role.

### 4.4. The Moderating Role of Social Activity

This study found that social activities play a protective role in the relationship between empathy and depression. When adolescents have low empathy abilities, they are less likely to develop depression if they can frequently participate in social activities. In the relationship between empathy ability and self-efficacy, social activities play a synergistic effect. Adolescents participating in frequent social activities can better leverage the advantages of high empathy abilities and more effectively improve self-efficacy. This shows the important position and role of social activities in the physical and mental development of adolescents. Researchers have found that social activities have a significant impact on adolescents’ brain cognitive functions, peer relationships, and distressed emotions [85,86,87]. The results of this study show that social activities have a protective effect on negative emotions and promote the improvement of self-efficacy, further validating the views of the above scholars.

### 4.5. Enlightenment and Shortcomings

Currently, there is very limited research on empathy and emotional resilience. This study is based on the overlap between the components of empathy and emotional resilience in order to study the influencing mechanisms of the two. This study enriches the relevant theories of empathy and emotional resilience and provides a valuable reference framework for subsequent research.

From a practical perspective, the results of this study have certain educational guidance significance. First, the impact of empathy on depression, self-efficacy, and emotional resilience reminds educators to pay attention to the cultivation of empathy in adolescents. Cultivating empathic thinking and practicing empathic behaviors can improve self-efficacy, reduce depression, reduce negative emotions, and improve emotional resilience in stressful situations, thereby promoting the mental health of teenagers. Secondly, the moderating effect of social activities on empathic depression and empathic self-efficacy shows that educators should provide support for adolescents’ social activities, avoid squeezing social activity time due to academic pressure, and encourage adolescents with insufficient social skills to participate in social activities to help and guide adolescents with social avoidance tendencies in adjusting themselves.

This study has certain limitations. First of all, in terms of sampling, this study selected samples from seven cities; however, the sample size was not evenly distributed in each city, and there may be a certain degree of sampling bias. Secondly, this study is a cross-sectional design, and it is impossible to draw causal conclusions based on the results. The causal relationship between variables needs to be further investigated and verified through experiments and follow-up studies to reveal the mechanism of action between variables. Thirdly, this study only studies all variables as a whole and does not study subdivided dimensions. Furthermore, this study neither identified other possible negative mediating relationships between empathy and emotional resilience nor explored the variables and mechanisms within the mediation model. Future investigation could build upon these gaps, delving into the complex mechanisms by which empathy influences emotional resilience.

In the future, positive empathy and negative empathy can be distinguished, and their impact mechanisms on emotional resilience can be examined separately to provide guidance for taking effective measures and more comprehensive and specific guidance for taking targeted youth training measures and experience reference. In addition, Zhang et al. [88] categorized empathy into emotional, cognitive, and behavioral empathy. Similarly, emotional resilience encompasses emotional, cognitive, and behavioral dimensions. Investigating the intricate connections between the dimensions of empathy and emotional resilience offers substantial potential for advancing our understanding in this field.

## 5. Conclusions

(1) The empathy of adolescents is positively correlated with emotional resilience. More empathy is associated with increased emotional resilience. (2) Depression serves as a complete mediator in the relationship between empathy and emotional resilience. Enhancing adolescents’ empathy can indirectly boost their emotional resilience by lowering depression levels. (3) Self-efficacy partially mediates the relationship between empathy and emotional resilience, additionally masking the direct effect of empathy and emotional resilience. (4) The relationship between empathy and depression is moderated by social activities. When adolescents have higher levels of social activity and empathy, the negative predictive effect of ability on depression is weakened. (5) The relationship between empathy and self-efficacy is moderated by social activities. When adolescents have higher levels of social activities, the positive predictive effect of empathy on self-efficacy is enhanced.

## Figures and Tables

**Table 1:** Mean number (M), standard deviation (SD), and correlation between the main variables (N = 1666).

	M	SD	Empathy	Depression	Self-Efficacy	Emotional Resilience	Social Activity
Empathy	52.100	9.032	1				
Depression	7.850	6.454	−0.142 ***	1			
Self-Efficacy	26.37	7.172	0.486 ***	−0.357 ***	1		
Emotional Resilience	40.28	10.739	0.103 ***	−0.635 ***	0.456 ***	1	
Social Activity	69.16	13.876	0.541 ***	−0.437 ***	0.549 ***	0.371 ***	1

Note: *** *p* < 0.001.

**Table 2:** Results of the analysis of the mediation effects of depression.

Prediction	M1 Emotional Resilience		M2 Depression		M3 Emotional Resilience	
	*β*	*t*	*β*	*t*	*β*	*t*
Age	−1.457 ***	−6.532	1.017 ***	7.535	−0.426 **	−2.378
Gender	−4.253 ***	−8.347	1.364 ***	4.425	−2.870 ***	−7.088
Only Child	−0.673	−1.227	0.086	0.258	−0.587	−1.353
Empathy	0.128 ***	4.580	−0.104 ***	−6.150	0.023	1.010
Depression					−1.014 ***	−31.648
R^2^	0.078		0.065		0.425	
F	34.895 **		28.925 ***		245.046 ***	

Note: *** *p* < 0.001; ** *p* < 0.01.

**Table 3:** Table of total effects, direct effects, and mediating effects of depression.

Mediator	Effect	Effect Value	Relative Effect Value	BootSE	BootLLCI	BootULCI
Depression	Total effect	0.128		0.028	0.073	0.183
	Direct effect	0.023		0.022	−0.021	0.067
	Mediating effect	0.106	82.030%	0.021	0.065	0.146

**Table 4:** The moderating role of social activity on empathy and depression.

Prediction	M7 Depression	
	*β*	*t*
Age	0.693 ***	5.606
Gender	0.968 ***	3.449
Only Child	0.064	0.214
Empathy	−0.153 **	−2.923
Social Activity	−0.405 ***	−10.151
Empathy * Social Activity	0.004 ***	4.791
R^2^	0.233	
F	84.179 ***	

Note: *** *p* < 0.001; ** *p* < 0.01; * *p* < 0.05.

**Table 5:** Mediating effect of self-efficacy.

Prediction	M4 Emotional Resilience		M5 Self-Efficacy		M6 Emotional Resilience	
	*β*	*t*	*β*	*t*	*β*	*t*
Age	−1.458 ***	−6.532	−0.959 ***	−7.286	−0.754 ***	−3.686
Gender	−4.252 ***	−8.347	−1.991 ***	−6.624	−2.791 ***	−5.997
Only Child	−0.673	−1.227	0.003	0.010	−0.676	−1.366
Empathy	0.128 ***	4.580	0.389 ***	23.521	−0.157 ***	−5.388
Self-Efficacy					0.734 ***	−19.580
R^2^	0.078		0.529		0.251	
F	34.895 ***		161.659 ***		111.019 ***	

Note: *** *p* < 0.001.

**Table 6:** Table of total effects, direct effects, and mediating effects of self-efficacy.

Mediator	Effect	Effect Value	Relative Effect Value	BootSE	BootLLCI	BootULCI
Self-efficacy	Total effect	0.128		0.028	0.073	0.183
	Direct effect	−0.157		0.029	−0.214	−0.100
	Mediating effect	0.286	234.44%	0.020	0.246	0.326

**Table 7:** The moderating role of social activity on empathy and self-efficacy.

Prediction	M8 Self-Efficacy	
	*β*	*t*
Age	−0.665 ***	−5.379
Gender	−1.552 ***	−5.534
Only Child	0.086	0.286
Empathy	0.055	1.040
Social Activity	0.059	1.468
Empathy* Social activity	0.003 ***	3.532
R^2^	0.380	
F	169.756 ***	

Note: *** *p* < 0.001.

## Data Availability

The datasets used and analyzed in the study are available from the corresponding author on request.
